# The prevalence and risk factors of acute kidney injury in post-orthopedic surgery patients

**DOI:** 10.3389/fmed.2026.1825528

**Published:** 2026-06-29

**Authors:** Wen Li, Chenfei Fu, Wenjing Fu, Aihua Zhang

**Affiliations:** 1 Department of Nephrology, Xuanwu Hospital, Capital Medical University, Beijing, China; 2 National Clinical Research Center for Geriatric Diseases, Xuanwu Hospital, Capital Medical University, Beijing, China

**Keywords:** acute kidney injury, orthopedic surgery, post-operative complications, prevalence, risk factors

## Abstract

**Purpose:**

This study aimed to investigate the prevalence and identify the risk factors associated with acute kidney injury (AKI) in patients undergoing orthopedic surgery.

**Methods:**

A retrospective study was conducted on 2,796 patients admitted to the orthopedic ward for bone tumors, bone injuries, and spinal diseases between 2017 and 2020. Patients were categorized into non-AKI, AKI stage 1–2, and AKI stage 3 groups. Data were analyzed using chi-square, Fisher’s exact, Kruskal-Wallis tests, and logistic regression to compare groups and determine independent risk factors for AKI.

**Results:**

AKI occurred in 97 patients, yielding an overall prevalence of 3.47%. The incidence was highest among patients undergoing bone tumor surgery (11.36%) and lowest in those undergoing spinal surgery (2.46%). Patients in the AKI stage 3 group had a significantly higher proportion of bone tumor surgeries and longer hospital stays compared to other groups (*P* < 0.001). Multivariate analysis identified bone tumor surgery, preoperative proteinuria, elevated urea, decreased hemoglobin, and the use of proton pump inhibitors (PPIs) and non-steroidal anti-inflammatory drugs (NSAIDs) as independent risk factors for AKI.

**Conclusion:**

There is a significant risk of AKI following orthopedic surgery, particularly in patients undergoing bone tumor procedures. The study underscores several key modifiable risk factors, including the use of PPIs and NSAIDs, as well as preoperative conditions like proteinuria, anemia, and elevated urea.

## 1 Introduction

Acute kidney injury is a common clinical complication characterized by a rapid decline in renal function in a short period of time. It is closely associated with high mortality, poor prognosis, and a variety of serious complications (1). In recent years, the prevalence of AKI has been increasing, posing a significant public health challenge. This trend is particularly concerning in perioperative patients, where AKI occurrence leads to prolonged hospital stays, increased medical costs, and a heightened risk of long-term adverse renal outcomes (2). Orthopedic surgery patients are at an elevated risk of AKI due to factors such as intraoperative blood loss, fluid imbalance, activation of inflammatory responses, and drug nephrotoxicity (3). AKI affects 5%–20% of patients after orthopedic surgery, with a recent large study reporting an incidence of 6.9% and an annual increase of approximately 17% (3, 4). Despite the existing focus on AKI following cardiac and gastrointestinal surgeries, there is a relative lack of systematic studies on the epidemiological characteristics and risk factors of AKI in orthopedic surgery patients. This study aims to investigate the prevalence of AKI in orthopedic surgery patients, analyze potential risk factors associated with AKI and its persistence, and provide an evidence-based approach for clinical prevention and treatment to reduce adverse outcomes related to AKI.

## 2 Objects and methods

### 2.1 Study subjects and methods

Patients hospitalized in the general ward of the Orthopedics Department at our hospital with bone tumors, bone trauma, and spinal diseases between 1 January 2017 and 31 December 2020, were enrolled in this study. The study protocol conformed to the principles of the Declaration of Helsinki. The study received approval from the Ethics Committee of Xuanwu Hospital, Capital Medical University [2022]228. Due to the retrospective nature of the study, patient information was anonymized, and the requirement for written informed consent was waived. Inclusion criteria encompassed patients aged 18 years and older who underwent surgery for bone tumors, bone trauma, and spinal diseases. Exclusion criteria included age < 18 years, preoperative AKI (defined as admission creatinine ≥ 1.5 times the MDRD-estimated baseline assuming a normal eGFR of 75 mL/min/1.73 m^2^), maintenance hemodialysis, and absence of paired preoperative and postoperative creatinine measurements within 1 week after surgery (5). Detailed demographic data, underlying diseases, laboratory test results, and pre- and post-surgery medication information were meticulously collected and analyzed. Anesthetic techniques were categorized into general anesthesia, spinal/epidural anesthesia, and peripheral nerve blocks based on anesthesia records.

### 2.2 Outcome measures

The primary outcome measure of this study was acute kidney injury (AKI), diagnosed and classified according to the 2012 Kidney Disease: Improving Global Outcomes (KDIGO) criteria (6). AKI stages were defined as follows: Stage I involved an increase in serum creatinine to 1.5–1.9 times the baseline value or an increase of 0.3 mg per deciliter (≥26.5 μmol per liter); Stage II indicated that serum creatinine had risen to 2.0–2.9 times the baseline value; Stage III denoted that serum creatinine had increased to 3.0 times the baseline value or higher, initiation of renal replacement therapy, or serum creatinine ≥ 4.0 mg/dl (≥353.6 μmol/L). Patients were initially classified into AKI and non-AKI groups based on their serum creatinine values during hospitalization. Within the AKI group, further subdivision was made into stages 1–2 and stage 3 for detailed analysis. For descriptive purposes, AKI was also classified by its duration as either transient (recovery within 48 h) or persistent (lasting ≥ 48 h) (7). Renal function was assessed using estimated glomerular filtration rate (eGFR), with preoperative eGFR calculated from baseline serum creatinine and postoperative eGFR from peak serum creatinine within 7 days post-surgery.

### 2.3 Statistical analysis

R4.3.2 software was utilized for data analysis. Normal distribution and homogeneity of variance were considered when presenting measurement data as mean ± standard deviation, while non-normally distributed data were displayed as median (P25, P75). Count data were represented as percentages or constituent ratios. Group comparisons are conducted using the chi-square test or Fisher’s exact test (for categorical data) and the Kruskal-Wallis test (for continuous data). Logistic regression analysis was conducted to identify the risk factors associated with acute kidney injury in patients undergoing orthopedic surgery. *P*-values less than 0.05 are considered statistically significant.

## 3 Result

### 3.1 Study population and screening

**FIGURE 1:**
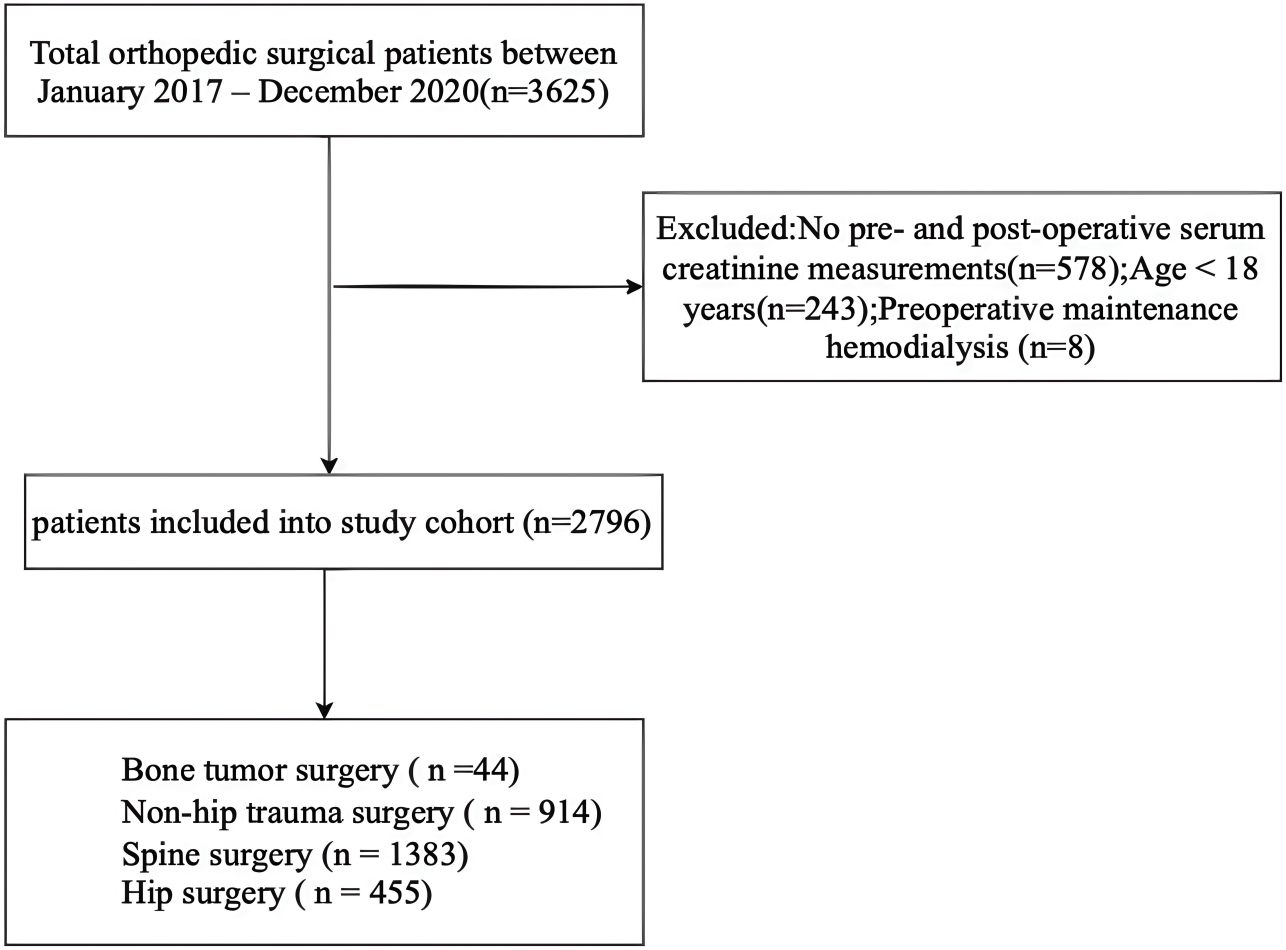
Patient selection flowchart.

A total of 3,625 patients who were hospitalized and underwent surgery in the general ward of the Department of Orthopedics at our hospital between 1 January 2017 and 31 December 2020, were initially identified. After excluding 578 patients who did not complete two serum creatinine tests before and within 1 week after surgery, 243 patients under 18 years old, and eight patients who were on maintenance hemodialysis before surgery, a final cohort of 2,796 patients were selected as the study subjects. The provided flow chart (Figure 1) illustrates the patient selection process.

### 3.2 Baseline clinical characteristics of the study cohort

A total of 2,796 patients were enrolled in this study, of which 97 patients (3.47%) had postoperative AKI, 40 patients (1.4%) had AKI stage 1–2, and 57 patients (2.0%) had AKI stage 3. In this study, 914 patients had non-hip surgery due to trauma, 1,383 patients underwent spinal surgery, 455 patients had hip surgery, and 44 patients had bone tumor surgery. None of the patients in the bone tumor group had received neoadjuvant therapy. The average age was 61 years. The average operation time was 2.37 h, and the average hospital stay was 9.59 days. A total of 551 patients (19.71%) had a history of diabetes, 910 patients (32.55%) had a history of hypertension, 672 patients (24.03%) had preoperative proteinuria, and 1,206 patients (43.13%) had heart failure. During hospitalization, 2,641 patients (94.46%) received cephalosporins, 1,148 patients (41.06%) were administered only proton pump inhibitors (PPIs), 303 patients (10.84%) received only NSAIDs, and 1,195 patients (42.74%) were treated with a combination of both NSAIDs and PPIs. Angiotensin II receptor blockers (ARBs) were prescribed to 314 patients (11.23%), and an iodine contrast agent was used in 11 patients (0.39%). There were significant differences among the three groups (non-AKI, AKI 1–2 stage, and AKI 3 stage) in terms of demographics, underlying diseases, preoperative laboratory data, and medication, as shown in Table 1.

**TABLE 1:** Patient characteristics and perioperative data by AKI stage.

Characteristic	Non-AKI group (*n* = 2,699)	AKI stage 1–2 group (*n* = 40)	AKI stage 3 group (*n* = 57)	Overall (*n* = 2796)	F/χ^2^/H	*P*-value
Surgical type [*n* (%)]					Fisher’s exact	<0.001*
Bone tumor surgery	39 (1.44)	1 (2.50)	4 (7.02)	44(1.57)	–	–
Spinal surgery	1,349 (49.98)	9 (22.50)	25 (43.86)	1,383(49.46)	–	–
Hip surgery	420 (15.56)	15 (37.50)	20 (35.09)	455(16.27)	–	–
Non-hip surgery due to trauma	891 (33.01)	15 (37.50)	8 (14.04)	914(32.70)	–	–
Length of stay (days)	8.00 (6.00, 11.00)	10.00 (8.00, 14.00)	10.00 (6.50, 15.00)	8.00 (6.00, 11.00)	20.866	0.001*
Operation time (h)	2.00 (1.00, 3.00)	2.00 (1.00, 3.50)	2.00 (1.00, 4.00)	2.00 (1.00, 3.00)	2.372	0.305
Age (years)	60.95 ± 16.44	68.48 ± 18.40	72.77 ± 16.31	61.30 ± 16.56	18.242	< 0.001*
Male [*n* (%)]	1,306 (48.39)	13 (32.50)	25 (43.86)	1,344 (48.07)	Fisher’s exact	0.074
BMI (Kg/m^2^)	24.84 ± 3.82	23.60 ± 2.84	24.78 ± 5.16	24.82 ± 3.87	2.017	0.133
SBP (mmHg)	130.00 (120.00, 140.00)	133.00 (130.00, 141.00)	140.00 (130.00, 142.00)	130.00 (120.00, 140.00)	11.763	0.003*
DBP (mmHg)	78.50 (70.00, 80.00)	68.00 (60.00, 81.00)	80.00 (78.25, 82.50)	79.00 (70.00, 80.00)	0.808	0.668
Hypertension [*n* (%)]	878 (32.53)	10 (25.00)	22 (38.60)	910 (32.55)	Fisher’s exact	0.370
Diabetes [*n* (%)]	518 (19.19)	12 (30.00)	21 (36.84)	551 (19.71)	Fisher’s exact	<0.001*
Heart Failure [*n* (%)]	1,158 (42.90)	19 (47.50)	29 (50.88)	1,206 (43.13)	1.762	0.414
Proteinuria positive [*n* (%)]	631 (23.38)	15 (37.50)	26 (45.61)	672(24.03)	Fisher’s exact	<0.001*
Hemoglobin (g/L)	131.20 ± 18.98	113.48 ± 22.84	120.25 ± 23.21	130.72 ± 19.30	36.057	<0.001*
Pre-op SCr (μmol/L)	59.00 (50.00, 70.00)	48.50 (39.00, 65.50)	76.00 (65.00, 88.00)	59.00 (50.00, 70.00)	48.417	<0.001*
Urea (mmol/L)	5.31 (4.37, 6.64)	6.19 (5.21, 7.50)	7.45 (5.15, 8.80)	5.35 (4.38, 6.70)	30.630	<0.001*
Uric acid (μmol/L)	322.41 ± 97.09	294.77 ± 111.44	374.76 ± 94.53	323.04 ± 97.55	7.291	<0.001*
Albumin (g/L)	39.13 ± 4.28	34.62 ± 5.31	37.77 ± 4.95	39.03 ± 4.35	24.087	<0.001*
Total cholesterol (mmol/L)	4.31 ± 0.97	3.91 ± 0.95	4.22 ± 1.45	4.30 ± 0.98	3.540	0.029*
Triglycerides (mmol/L)	1.21 (0.86, 1.71)	1.09 (0.85, 1.55)	1.03 (0.76, 1.61)	1.21 (0.86, 1.71)	1.047	0.593
LDL-C (mmol/L)	2.65 ± 0.82	2.22 ± 0.67	2.59 ± 1.18	2.64 ± 0.83	4.120	0.016*
HDL-C (mmol/L)	1.23 ± 0.33	1.18 ± 0.32	1.22 ± 0.38	1.23 ± 0.33	0.418	0.659
Fibrinogen (g/L)	3.78 (3.20, 4.67)	4.73 (3.97, 5.49)	4.68 (3.82, 5.03)	3.33 (2.87, 4.01)	11.550	0.003*
HbA1c (%)	6.30 (5.60, 7.30)	7.15 (5.20, 9.10)	6.45 (5.80, 8.90)	6.30 (5.60, 7.20)	0.178	0.915
Leukocyte (10^9^/L)	6.64 (5.42, 8.26)	7.27 (6.09, 9.99)	7.05 (5.38, 9.16)	6.66 (5.42, 8.29)	5.57	0.062
PaO_2_ (mmHg)	76.85 (70.90, 85.50)	68.15 (55.60, 80.70)	78.00 (63.90, 86.50)	79.40 (71.88, 88.90)	1.779	0.411
SpO_2_ (%)	95.60 (93.80, 96.80)	90.90 (86.40, 95.40)	96.00 (94.50, 97.20)	96.00 (94.60, 97.10)	3.892	0.143
Anesthetic technique						
General anesthesia (%)	1,536 (56.9%)	40 (41.2%)	22 (38.6%)	1,598 (57.2%)	2.910	0.088
Peripheral nerve block (%)	814 (30.2%)	16 (16.5%)	6 (10.5%)	836 (29.9%)	0.310	0.578
Spinal/epidural anesthesia (%)	446 (16.5%)	9 (9.3%)	4 (7.0%)	459 (16.4%)	1.705	0.191
Blood loss (mL)	100.00 (50.00, 200.00)	150.00 (80.00, 250.00)	220.00 (120.00, 250.00)	100.00 (50.00, 250.00)	4.269	0.119
Transfusion rate (%)	386 (14.30)	5 (12.50)	9 (15.79)	400 (14.31)	Fisher’s	0.895
Medicine use						
Cephalosporin use [*n* (%)]	2,546 (94.33)	39 (97.50)	56 (98.25)	2,641(94.46)	Fisher’s exact	0.309
ARB use [*n* (%)]	296 (10.97)	5 (12.50)	13 (22.81)	314 (11.23)	Fisher’s exact	0.019*
NSAID with PPI [*n* (%)]	1,134 (42.02)	25 (62.50)	36 (63.16)	1,195 (42.74)	Fisher’s	<0.001*
PPI use only [*n* (%)]	1,121 (41.53)	12(30.00)	15 (26.32)	1,148 (41.06)	Fisher’s exact	0.025*
NSAID use only [*n* (%)]	300 (11.12)	1 (2.50)	2 (3.50)	303 (10.84)	Fisher’s	0.044*
Contrast use [*n* (%)]	10 (0.37)	0 (0.00)	1 (1.75)	11 (0.39)	Fisher’s exact	0.236

*P-values less than 0.05 are considered statistically significant. AKI, acute kidney injury; BMI, body mass index; SBP, systolic blood pressure; DBP, diastolic blood pressure; Pre-op SCr, pre-operative serum creatinine; LDL-C, low-density lipoprotein cholesterol; HDL-C, high-density lipoprotein cholesterol; PaO2, partial pressure of oxygen in arterial blood; SpO2, partial pressure of oxygen in arterial blood; NSAID, non-steroidal anti-inflammatory drug; ACEI/ARB, angiotensin-converting enzyme inhibitor/angiotensin II receptor antagonist; PPI, proton pump inhibitor.

Age was found to increase significantly with the severity of AKI (non-AKI: 60.95 ± 16.44 years; AKI stage 1–2: 68.48 ± 18.40 years; AKI stage 3: 72.77 ± 16.31 years, *P* < 0.001). The percentage of patients with diabetes, proteinuria, and preoperative systolic blood pressure also demonstrated a significant increase with the severity of AKI (*P* < 0.001). Different types of surgery were significantly associated with the prevalence and severity of AKI (*P* < 0.001). Among patients undergoing bone tumor surgery, the highest proportion of AKI cases was observed in the AKI stage 3 group (7.02%, 4/57).

### 3.3 Preoperative laboratory parameters and medication use across non-AKI, AKI stage 1–2, and AKI stage 3 patient groups

The differences in preoperative serum creatinine, blood urea nitrogen, uric acid, albumin, total cholesterol, LDL-C, and fibrinogen levels among the three groups were statistically significant. Regarding medication usage, the proportions of NSAIDs, ARB drugs, and PPI drugs were higher in the AKI group than in the non-AKI group (*P* < 0.05).

### 3.4 Operative time and length of hospital stay across non-AKI, AKI stage 1–2, and AKI stage 3 patient groups

The median hospital stay for the AKI group was 10 days, which was significantly longer than that for the non-AKI group (8 days) (*P* = 0.002). However, there was no significant difference in operation time between the three groups (*P* = 0.125).

### 3.5 Incidence and severity of postoperative eGFR deterioration

We further analyzed the deterioration of eGFR, and a total of 1,182 patients (42.3%) had some degree of deterioration in eGFR from preoperative baseline. Among these patients, 8.2% exhibited a 20%–29% reduction in eGFR from baseline (*n* = 97), 2.9% showed a 30%–39% reduction in eGFR from baseline (*n* = 34), 0.8% had a 40%–49% reduction in eGFR from baseline (*n* = 9), and 1.3% experienced a ≥ 50% reduction in eGFR (*n* = 15), as presented in Table 2.

**TABLE 2:** Changes in eGFR after orthopedic surgery.

Category	Patients, *n* (%)
No deterioration compared to baseline (%)	1,614 (57.7%)
Deterioration compared to baseline (%)	1,182 (42.3%)
eGFR decrease 20–29	97 (8.2%)
eGFR decrease 30–39	34 (2.9%)
eGFR decrease 40–49	9 (0.8%)
eGFR decrease ≥ 50	15 (1.3%)

Data are presented as the number of patients (%). The total cohort consisted of 2,796 patients. Deterioration is defined as a decrease in eGFR from baseline. eGFR, estimated Glomerular Filtration Rate.

### 3.6 Risk factors for AKI patients after undergoing orthopedic surgery

Univariate logistic regression analysis revealed that hip surgery and bone tumor surgery were significant risk factors for AKI in orthopedic surgery patients (*P* < 0.05), with the incidence of AKI after vertebral surgery as the reference. Other identified risk factors included advanced age, preoperative diabetes mellitus, preoperative proteinuria, elevated serum creatinine, elevated blood urea nitrogen, elevated fibrinogen, decreased hemoglobin, decreased serum albumin, decreased total cholesterol, decreased low-density lipoprotein cholesterol, the use of ARB drugs, PPI monotherapy, NSAID monotherapy, and combined PPI and NSAID therapy (*P* < 0.05).

**FIGURE 2:**
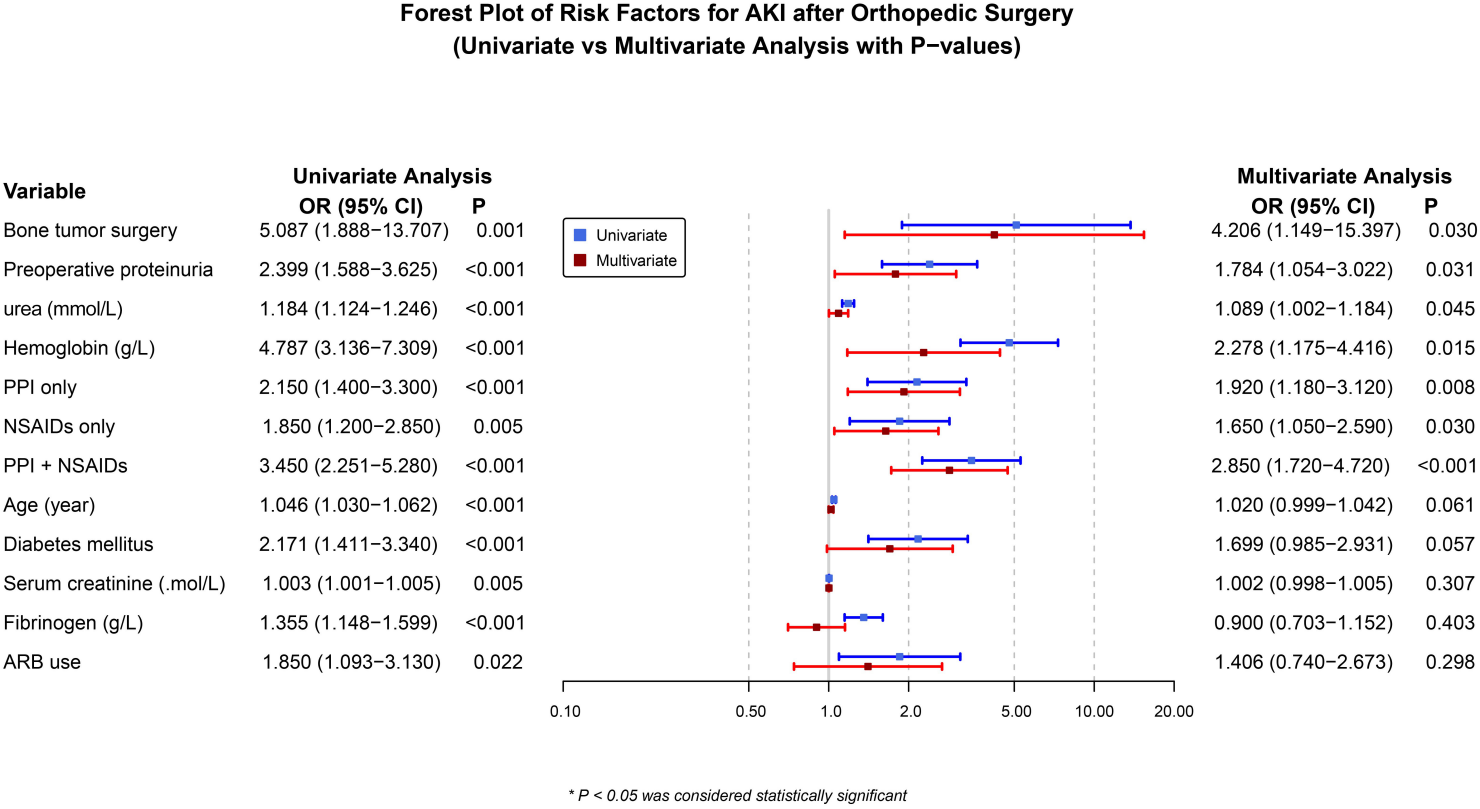
Univariate and multivariate Logistic regression analysis of risk factors for AKI. Surgical type and medication use are presented as categorical variables [*n* (%)], while other variables are continuous. *P*-values less than 0.05 are considered statistically significant (*). Parameters with *P* < 0.1 in univariate logistic regression were included in multivariate analysis. OR, odds ratio; 95% CI, 95% confidence interval; P, statistical significance level; AKI: acute kidney injury; ARB: angiotensin II receptor antagonist; PPI, proton pump inhibitor; NSAID, non-steroidal anti-inflammatory drug.

Parameters with *P* < 0.1 in univariate logistic regression were included in multivariate analysis. In the multivariate logistic regression analysis, independent risk factors for AKI in patients undergoing orthopedic surgery were identified as follows: bone tumor surgery, preoperative proteinuria, elevated blood urea nitrogen, anemia, use of PPI monotherapy, use of NSAID monotherapy, and combined PPI-NSAID therapy (P < 0.05). The results are presented in Figure 2.

### 3.7 Clinical course of post-operative (persistent AKI or transient AKI)

In this study, a total of 97 patients who developed acute kidney injury (AKI) following orthopedic surgery were included. After excluding 39 cases with incomplete clinical data, which made it impossible to define persistent or transient AKI,58 patients were retained for analysis. Among this group, 17 cases (29.3%) were identified as having persistent AKI, while 41 cases (70.7%) were classified as transient AKI. Within the subgroup of patients with persistent AKI, two individuals (11.8%) required renal replacement therapy.

## 4 Discussion

Previous studies have reported different incidence rates of postoperative AKI ranging from 6.8% to 10% in orthopedic cohorts (3, 8, 9). However, these studies often had limited sample sizes (fewer than 1,000 cases) and focused on a single surgical procedure. In our large-sample study encompassing various orthopedic procedures, we found that the overall prevalence of postoperative AKI was 3.47%. Moreover, the risk of AKI significantly varied among different surgical procedures, with bone tumor surgery having the highest risk (11.36%), followed by hip surgery (7.69%), and the lowest risk observed during spine surgery (2.46%). In addition, our study revealed that approximately 29.3% of AKI patients developed persistent AKI (lasting ≥ 48 h), hints associated with a poorer clinical prognosis as previous publications (10, 11).

The etiology of AKI following orthopedic surgery is complex, which may be related to the operation itself and the accompanying strong physiological stress, including intraoperative hemodynamic fluctuations, inflammatory response activation, potential bone cement implantation syndrome (12–14).

This study replicated previous findings that preoperative proteinuria, elevated blood urea nitrogen level, use of non-steroidal anti-inflammatory drugs, and anemia are significant risk factors for postoperative AKI (15–19). After adjusting for confounding factors, our study revealed that preoperative use of PPIs, as well as the combined use of PPIs and NSAIDs, is an independent risk factor for the development of AKI following orthopedic surgery. Several mechanisms might be involved. The main effect of PPIs on the kidney is to cause acute interstitial nephritis (AIN), the pathogenesis of which remains unclear but may involve the deposition of PPIs and their metabolites in the renal tubulointerstitium, acting as haptens or directly stimulating T cell expression and the CYP2C19 genotype (20). Additionally, PPIs can induce renal dysfunction by reducing tubular cell regeneration, increasing oxidative stress, and altering gene expression (21). Our findings align with the existing literature, which has demonstrated a connection between PPI use and postoperative AKI in patients undergoing major surgical procedures and cardiac surgery (22–25). Furthermore, the combined use of PPIs and NSAIDs has been associated with an increased risk of AKI, as both agents can independently and synergistically contribute to renal injury through mechanisms such as impaired renal blood flow, tubulointerstitial inflammation, Drug metabolism interactions, and oxidative damage (26).

In our study, the occurrence rate of AKI in patients undergoing bone tumor surgery was notably high at 11.36%, exceeding the risk associated with general orthopedic procedures by more than threefold. Furthermore, 7.02% of AKI cases in this group progressed to AKI stage 3. The elevated occurrence and severity of AKI can be attributed to a confluence of factors unique to bone tumor surgeries compared to other orthopedic procedures. Bone tumor surgery is typically more extensive, with longer operation times and greater complexity, which may involve large vessel manipulation or wide resection (27). Meanwhile, in our cohort, none of the patients received preoperative neoadjuvant therapy, highlighting those other mechanisms, rather than chemotherapy, also play a significant role in the development of AKI. Malignant bone tumors are known to release proinflammatory cytokines and other mediators that contribute to a systemic inflammatory response. Additionally, they can induce metabolic disturbances, such as hypercalcemia, which directly impair renal function (28). Surgical site infections (SSIs), which are more commonly observed in patients undergoing bone tumor surgery compared to other orthopedic procedures, represent another significant risk factor for AKI. SSIs can trigger a systemic inflammatory response syndrome (SIRS), leading to hemodynamic instability and reduced renal perfusion (29). Furthermore, infections can directly damage renal tissue, further exacerbating renal injury. These patients may also exhibit poorer overall nutritional status and baseline health conditions, further predisposing them to AKI (30). The collective impact of these factors suggests bone tumor surgery as a high-risk procedure for the development of AKI, especially severe AKI.

Previous studies have suggested that intraoperative factors such as anesthesia techniques, blood loss, and transfusion may contribute to acute kidney injury (AKI). However, our analysis did not find a significant association between these factors and AKI, consistent with evidence from other non-cardiac surgical settings (31, 32). Recent research in cardiac surgery suggests that the relationship between blood loss and AKI may be mediated by hemodynamic instability, rather than direct renal injury (33). In our cohort of orthopedic patients, no cases of major hemodynamic instability were observed, likely due to our study population being drawn from general wards, rather than the higher-risk ICU patients. These findings underscore the need for further research on intraoperative hemodynamic management and fluid resuscitation in preventing AKI in non-cardiac surgeries. Future studies should focus on orthopedic surgery patients, particularly those in the ICU, to explore the role of precise hemodynamic control and fluid management in reducing postoperative AKI.

Several other factors that may influence AKI risk were examined. First, no significant association was observed between heart failure and postoperative AKI, such as Mathis et al. reported that accurately diagnosed heart failure was associated with a 61% lower risk of postoperative AKI (34). In our cohort, most heart failure patients had well-compensated HFpEF (NYHA Class I–II) and all underwent rigorous preoperative multidisciplinary evaluation, including anesthesiology assessment, orthopedic evaluation, and specialist consultation when indicated, to ensure optimal clinical status before surgery. Second, active infection did not substantially confound our findings: white blood cell count showed no significant difference among AKI groups; patients with preoperative fever or infectious leukocytosis were deferred from elective surgery; and no primary orthopedic infectious diseases or infection-related discharge diagnoses were documented. Third, preoperative renal imaging was performed in only 5.5% of patients, with only three cases of structural abnormalities identified. In the future, we encourage orthopedic surgeons to consider incorporating renal ultrasound into postoperative assessment protocols for high-risk patients and to establish closer collaboration with nephrology departments to improve perioperative kidney care.

This study has confirmed that AKI significantly prolongs hospital stay in patients undergoing orthopedic surgery (35). This observation aligns with extensive literature documenting that AKI is independently associated with increased healthcare costs, heightened risks of in-hospital complications, and elevated both short- and long-term mortality (1, 14, 36). Our data, showing a markedly longer median hospital stay for AKI patients, thus underscores the substantial clinical and economic burden imposed by this postoperative complication.

To decrease AKI prevalence, it’s crucial to conduct routine risk assessments for orthopedic surgery patients, especially those undergoing bone tumor procedures. Preoperative assessments like screening for proteinuria, monitoring urea levels, reviewing medication history, and addressing anemia are essential. Enhanced renal function monitoring during the perioperative period is critical (37). Evaluate the use of PPIs and NSAIDs carefully, considering H2-receptor blockers for AKI events requiring ongoing acid-suppressive therapy (38, 39). In the postoperative period, the risks and benefits of continued PPI use should be carefully weighed for each patient (40, 41).

This study also presents certain limitations. Firstly, being a retrospective single-center survey may introduce bias. Secondly, certain intraoperative parameters were not fully captured, potentially impacting the assessment of AKI occurrence. Only patients with postoperative serum creatinine monitoring were included, potentially excluding mild or asymptomatic AKI cases in those not observed. Finally, the observations were confined to the hospitalization period, preventing an assessment of the long-term prognosis of AKI.

Despite these limitations, our study offers valuable insights into the incidence and progression of AKI in a large cohort of patients undergoing orthopedic surgery. Larger, multicenter, prospective studies in the future are essential for further confirmation.

## Data Availability

The raw data supporting the conclusions of this article will be made available by the authors, without undue reservation.
